# Two Cases of Syphilis, in Which the Infection Took Place in Rather Unusual Situations

**Published:** 1878-07

**Authors:** George H. Rohé

**Affiliations:** Lecturer on Diseases of the Skin, College of Physicians and Surgeons, Baltimore


### TWO CASES OF SYPHILIS, IN WHICH THE INFECTION TOOK PLACE IN RATHER UNUSUAL SITUATIONS.

By George H. Rohé, M. D.

[*Lecturer on Diseases of the Skin, College of Physicians and Surgeons, Baltimore.*)

In the current (July) number of the *Archives of Dermatology*, I have reported a case where syphilis had been communicated by a bite on the nose, inflicted during a fight. Since reporting that case, two others have come under my observation, which appear to me of sufficient interest to warrant publication.

Case I. *Chancre of the Tongue.* C. K., white, American, widow, 27 years of age, and by occupation factory operative, consulted me on the 24th of May, 1878, with the following history:

About the middle of last February she noticed a whitish pimple on the surface of the tongue, near its tip, which was slightly painful. This pimple became larger, hard at the base, and eventually resulted in an ulcer. Up to this time the only application she had used was a mouth-wash of borax, which seemed however to do no good. She then consulted a physician, who cauterized the lingual ulcer several times, and gave her some pills which produced salivation. The tongue became greatly swollen and painful, causing considerable trouble in eating and speaking. Shortly afterward, her throat became very sore, and she was sick and feverish, being compelled to take to her bed for a few days in consequence. About three weeks ago, while dressing, she discovered a brownish rash upon her body, and consulted me with regard to it, on the date mentioned above. Her husband died over a year ago, of some phthisical affection. He never had any cutaneous eruption, to her knowledge. Two children died soon after birth, of "brain trouble;" both had sore eyes and "a wheezing" in the throat, but no skin disease. One child, the eldest, eight years old, is living, who is deficient in mental capacity, but otherwise appears healthy.

*Present condition.*—Her face, arms and body are thickly covered with reddish, or brownish-yellow, flat papules, the summits of which are slightly scaly. Upon the face, the papules are localized especially about the angles formed by the alæ of the nose with the cheeks, and the angles of the mouth. The submaxillary glands are much enlarged and tender; the post-cervical only slightly so. On examining the mouth, an oblong, slightly excavated patch, with a deep-brown glossy base, is seen near the tip of the tongue, occupying the middle line of the organ. The base is firmly infiltrated, the induration being rather limited in extent. The throat is still red, but not painful. The inguinal glands are very slightly enlarged, but not indurated or tender. An examination of the genitals reveals no lesion or induration of the vulva or vaginal mucous membrane. The anterior segment of the os uteri is perfectly normal to the touch, while the posterior segment is firm and hard, conveying to the finger the sensation of the induration of a cancerous infiltration, or a firm cicatrix, more nearly than anything else I can compare it to. The os is normal in size, and neither painful nor tender to the touch. She does not remember having any sore or other trouble about her genital organs recently; and absolutely denies any sexual intercourse since the death of her husband, which, as stated above, occurred over a year ago.

None of the other operatives, in the factory where she works, had any sore mouth or skin affection, to her knowledge, but a young man who visited frequently at her brother's house, and whom she occasionally met there, had a copious eruption on his face. She remembered that on one occasion they had some sort of game, consisting in writing little notes to one another and passing them around the room. The young man referred to was present and took part in the game. A pencil belonging to him was used and passed around, but she has no recollection of putting the pencil in her mouth. It was some time after this that the pimple appeared on her tongue. The eruption on the skin was characteristic and sufficient to justify the diagnosis of syphilis, and the regular consecutive appearance of the other symptoms following the affection on the tongue, led me to believe the latter to be the primary lesion, which belief was strengthened by the negative result of the examination of the genitals. The induration of the posterior segment of the os uteri I believe to be either cicatricial or cancerous. She was put upon inunction with mercurial ointment, after Sigmund's plan, and directed to take fifteen drops of tincture of chloride of iron three times a day. Under this treatment the eruption rapidly faded, and has at the time of writing nearly disappeared.

Case II. J. S., American, male, 24, shoemaker; came to my clinic, at the College of Physicians and Surgeons, on May 30, 1878, with the following history: About six weeks ago a sore came upon his upper lip, which became much swollen ; about a month later his throat became very sore, and about a week ago he noticed an eruption upon his body. His present condition is as follows: The face, body and extremities are covered by a copious erythematous syphiloderm. The upper part of the chest, face and back of neck are also thinly studded with papules. On the upper lip, a little to the left of the middle line, is a swelling about the size of a pigeon's egg, with the mucous membrane everted and excoriated; the submucous tissue is the seat of a thin, firm infiltration distinctly circumscribed. The submaxillary glands are very much enlarged and tender; post-cervical glands also enlarged; inguinal glands very slightly enlarged, not tender to the touch. The mucous layer of the prepuce and the glands is the seat of five or six small, lenticular excoriations, without induration, which look like papules of the mucous membrane deprived of their epithelial covering. These spots had existed only four or five days. He has not had sexual intercourse for over four months. Denies having ever had any affection of the genital organs, either ulcerative or blennorrhœal. He remembers kissing a girl having a sore lip, about two months ago.

The evolution of the disease in its various stages in this case, is entirely consistent with the assumption that the sore on the lip was the initial lesion, and that this was the result of inoculation during the suspicious kiss. It is to be regretted that there could not be confrontation in this case to decide the question.

He was ordered mercurial inunction, and calomel to be dusted on the lip. As he did not return to the clinic, the result of treatment could not be followed up.

